# Phenobarbital and Alcohol Withdrawal Syndrome: A Systematic Review and Meta-Analysis

**DOI:** 10.7759/cureus.33695

**Published:** 2023-01-12

**Authors:** Zaryab Umar, Muhammad Haseeb ul Rasool, Shoaib Muhammad, Sara Yousaf, Mahmoud Nassar, Usman Ilyas, Asma U Hosna, Avish Parikh, Rubal Bhangal, Nazaakat Ahmed, Jonathan Ariyaratnam, Theo Trandafirescu

**Affiliations:** 1 Internal Medicine, Icahn School of Medicine at Mount Sinai, Queens Hospital Center, New York City, USA; 2 Medicine, Icahn School of Medicine at Mount Sinai, Queens Hospital Center, New York City, USA; 3 Radiology, Salam Medical Complex, Lahore, PAK; 4 Urology, Gulab Devi Hospital, Al-Aleem Medical College, Lahore, PAK; 5 Anesthesiology and Intensive Therapy Unit, Dartford and Gravesham NHS Trust, Dartford, GBR

**Keywords:** intratracheal intubation, length of stay in icu, icu patients, phenobarbital therapy, alcohol withdrawal syndrome

## Abstract

Alcohol withdrawal syndrome (AWS) is a complication frequently encountered among patients who are chronic alcohol abusers. It is considered to have a significant impact on the United States healthcare system. It not only has a toll on the healthcare spending but also contributes to significant morbidity and mortality. Benzodiazepines are considered first line in the treatment of AWS. Since patients with alcohol use disorder have downregulated gamma aminobutyric acid (GABA) receptors, this often leads to benzodiazepine resistance. Phenobarbital is also used in the management of alcohol withdrawal syndrome. Here we present a systematic review and meta-analysis of the efficacy and safety of the drug.

We conducted an electronic database search for relevant studies published between the inception of the project and November 20, 2022, in three databases, including Medline/PubMed, Embase, and Cochrane Library. Our study included all original studies with prime focus on the baseline characteristics of patients admitted to the intensive care unit (ICU) for alcohol withdrawal syndrome and management/monitoring protocol implemented for its treatment. The primary outcomes that were the focus of our study consisted of changes in the length of hospital stay, length of ICU stay, and changes in scoring systems (for alcohol withdrawal assessment and monitoring) following the implementation of phenobarbital. The secondary outcomes included complications such as intubation and mortality.

Based on our analysis, the mean difference in hospital stay was statistically significant at -2.6 (95% CI, -4.48, -0.72, P=0.007) for phenobarbital compared to the benzodiazepine group. We were unable to comment on the heterogeneity in our meta-analysis due to the standard deviation not being reported in one study. There was no statistically significant difference regarding the length of stay in the intensive care unit compared to the control/comparative arm, with a mean difference of -1.17 (95% CI, -1.17, 0.09, P=0.07), with considerable heterogeneity (I^2^=77%, P=0.002). Our meta-analysis also investigated the risk of intubation between the phenobarbital and the control/comparative group. There was statistically significant difference in the incidence of intubation, relative risk (RR) 0.52 (95% CI, 0.25, 1.08, P=0.08), with considerable heterogeneity (I^2^=80%, P=0.0001).

Our study concludes that phenobarbital is an effective tool in the management of AWS in an ICU setting. However, various studies have reported contradictory results, and vital information appears to be lacking. Moreover, there is a lack of uniformity in terms of phenobarbital dosing. Drug administration should be adapted according to the severity of the symptoms. Further studies need to be conducted discussing the safety profile and adverse effects of the drug when it comes to the management of alcohol withdrawal syndrome.

## Introduction and background

In the United States, alcohol abuse represents a significant healthcare burden, with over 14 million Americans suffering from alcohol use disorder. In 2010, a total of $249 billion was spent in the United States on alcohol-related disorders. Despite warnings from the World Health Organization, alcohol consumption is expected to increase until at least 2025. Hospital admissions for chronic alcohol abusers are particularly dangerous since these patients are at an increased risk of developing life-threatening conditions such as alcohol withdrawal syndrome (AWS) [1].

Chronic alcoholism leads to a decrease in gamma aminobutyric acid (GABA) inhibitory receptors as part of a feedback mechanism to counteract the depressive effects of alcohol. Additionally, excitatory glutamate receptors are also upregulated, decreasing the influence of alcohol. When alcohol intake is abruptly ceased, the body does not have enough time to adjust GABA and glutamate regulation, resulting in alcohol withdrawal syndrome. Symptoms of AWS begin to manifest eight hours after the last drink and peak 24 to 72 hours later. Patients initially experience nausea, sweating, headaches, shakiness, tachycardia and hypertension. Symptoms become more severe over time, leading to seizures and delirium tremens [2,3].

Pharmacological agents such as benzodiazepines, barbiturates, beta-blockers, butyrophenones, gabapentin, propofol, dexmedetomidine, and valproic acid have been used to treat alcohol withdrawal syndrome to reduce its intensity and prevent life-threatening complications. The ideal agent for treating AWS should have a rapid onset, a strong safety profile and properties such as anxiolysis and sedation. The American Society of Addiction Medicine's 2020 Clinical Practice Guidelines recommend benzodiazepines as the first-line treatment for addiction [4]. The binding of benzodiazepines to GABA receptors increases the frequency of opening of these channels. Benzodiazepines are fully effective when GABA is available at the receptor site. A patient with alcohol use disorder has low levels of GABA, causing benzodiazepines to fail to produce the desired response, resulting in resistant alcohol withdrawal syndrome, characterized by hallucinations, seizures, and delirium tremens [3-5]. Therefore, other pharmacological agents can be used to control the debilitating symptoms of alcohol withdrawal syndrome. Phenobarbital (a barbiturate) is being increasingly recommended for the treatment of patients with a contraindication to benzodiazepines [4]. Phenobarbital also acts on glutamate in addition to GABA. GABA and glutamate receptors are both responsible for the symptoms of AWS. The effect of phenobarbital is robust because, unlike benzodiazepines, it does not require endogenous GABA [3,6,7]. Several observational studies have been published on the effectiveness of phenobarbital in AWS, with mixed results. As a result, we conducted our meta-analysis in order to assess phenobarbital's effectiveness in AWS.

## Review

Search strategy and study selection

The study methodology was based on the Preferred Reporting Items for Systematic Reviews and Meta-Analyses (PRISMA) guidelines, in which protocol registration is not required [8]. We conducted an electronic database search for relevant studies published between the inception of the project and November 20, 2022, in three databases, including Medline/PubMed, Embase, and Cochrane Library. In our search, we used keywords, Medical Subject Headings (MeSH) terms, and publication type based on the participants, comparison, intervention, and outcomes (PICO) framework. The following terms were used according to each database: "Barbiturate" OR "Barbi" OR "Barb" OR "Phenobarbital" OR "Phenobarb" OR "Pheno" OR "Phenobarbital"[Mesh] OR "Barbiturates"[Mesh] AND "Alcohol withdrawal syndrome" OR "Alcohol withdrawal" OR "Delirium tremens" OR "Delirium" OR "Alcohol Withdrawal Delirium"[Mesh] OR "Alcohol-Induced Disorders, Nervous System"[Mesh] OR "Substance Withdrawal Syndrome"[Mesh] OR "Alcoholism"[Mesh] AND "Intensive care" OR "Intensive care unit" OR "Critical care" OR "Intensive therapy unit" OR "Intensive Care Units"[Mesh].

The study included all original studies, including cohort, cross-sectional, and case-control studies, which provided information on baseline characteristics, comorbidities, initial assessment of patients with alcohol withdrawal syndrome, management using phenobarbital, and a monitoring protocol implemented in the intensive care unit (ICU) for the treatment of AWS, outcomes, and complications observed. In addition, we included commentary and case series that included at least 10 patients. Exclusion criteria included non-original reports, such as reviews, letters to editors, and commentaries, which did not include original patient data. In addition, case reports and case series involving fewer than 10 patients were excluded. This study excluded the following: full texts with limited data/data not relevant to the study, unextractable or irrelevant data, articles not published in English, duplicate records, animal studies, and overlapped data.

The primary outcomes were the mean length of hospital stay, mean length of ICU stay, and changes in Clinical Institute Withdrawal Assessment for Alcohol (CIWA) scale/additional scoring system scores following the implementation of phenobarbital. The secondary outcomes included complications (such as intubation) and mortality.

We conducted a manual search of the references of our included papers in order to ensure that we did not miss any relevant studies. All original studies that reported the management of AWS in the ICU setting with phenobarbital were included in the analysis. Two independent reviewers screened the titles and abstracts of the papers, followed by a full-text screening to ensure that relevant papers were included in the systematic review. Disagreements were resolved by discussion and by referring to the senior author whenever necessary.

Data extraction

The data extraction sheet was developed by one author using Microsoft Excel (Microsoft, Redmond, WA). Two independent reviewers extracted data using an Excel spreadsheet. The author who developed the data extraction sheet checked the extracted data for accuracy. When necessary, the senior author was consulted for the resolution of disagreements and discrepancies.

Quality assessment and statistical analysis

An independent reviewer evaluated the risk of bias in the included studies. We assessed the quality of the included studies using the risk-of-bias assessment tool developed by the National Institutes of Health (NIH) [9]. An analysis of the descriptive data was conducted using SPSS Statistics, version 26 (IBM Corp., Armonk, NY). RevMan 5.4 (Cochrane Collaboration, Copenhagen) was used to develop the forest plot and funnel plot.

Results

Search Results

**Figure 1:**
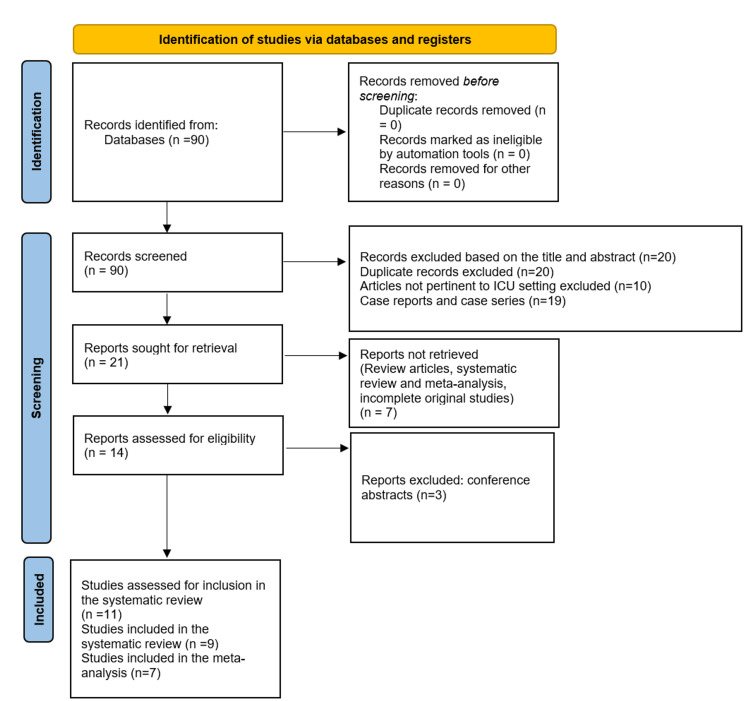
Flow diagram of the PRISMA screening process for this systematic review and meta-analysis PRISMA: Preferred Reporting Items for Systematic Reviews and Meta-Analyses

We identified 90 records using EndNote 20 (Clarivate, London). After removing duplicate records and manually screening abstracts and titles, 21 records were identified for further full-text screening. A total of 11 studies were assessed for inclusion into our systematic review. Two studies were excluded due to the data being individualized for every patient and lack of mean/median values of the variables examined for the entire study population. Ultimately, nine studies were included in this systematic review and meta-analysis after 12 papers were excluded from the full-text screening phase (Figure 1).

Study Characteristics and Quality of the Included Studies

The baseline characteristics of the included studies are summarized in Table 1. A total of nine studies were included: one retrospective case series with more than 11 participants and including original data [10], seven retrospective studies [11-17], and one abstract with data relevant to the study design [18]. The sample sizes of the included studies ranged from 31 to 135 individuals. Patients aged 18 and older were included in the study.

**Table 1:** Study characteristics n: number of patients; SD: standard deviation; SICU: surgical intensive care unit; AWS: alcohol withdrawal syndrome; AUD: alcohol use disorder; IV: intravenous; DTs: delirium tremens; MICU: medical intensive care unit; BZD: benzodiazepine; APACHE II: Acute Physiology and Chronic Health Evaluation II; CIWA: Clinical Institute Withdrawal Assessment for Alcohol; RASS: Richmond Agitation and Sedation Scale; CAM-ICU: Confusion Assessment Method for ICU; mMINDS: modified Minnesota Detoxification Scale; MELD: Model for End-stage Liver Disease; SOFA: Sequential Organ Failure Assessment ^a^Patients with a high risk for AWS were defined as patients with a previous history of DTs with or without history of alcohol withdrawal seizures and recent alcohol use (≤2 weeks) with positive blood alcohol levels (BALs) >0.1 g/dL, or elevated mean corpuscular volume (MCV), or elevated aspartate aminotransferase (AST) to alanine aminotransferase (ALT) ratio ≥1.5:1. Patients with a medium risk for AWS were defined as patients with active alcohol dependence per patient's reporting plus two of the following criteria: two or more days since last drink, or an elevated BAL >0.1 g/dL on admission, or symptoms of autonomic dysfunction with the BAL >0.1 g/dL, or elevated MCV, or AST:ALT ratio ≥1.5:1.28-31. <> denotes not mentioned/not available.

Authors	Year	Study groups		Age, mean ± SD/median (interquartile range)		Total patients, n (%)		Male, n/%		Prior history/comorbidities, n/%		Screening/assessment tool for AWS	
		Intervention	Control/comparative arm	Intervention	Control/comparative arm	Intervention	Control/comparative arm	Intervention	Control/comparative arm	Intervention	Control/comparative arm	Intervention	Control/comparative arm
Ammar et al. (retrospective case series) [10]	2020	All patients in SICU with AWS who received phenobarbital monotherapy for the prevention or worsening of AWS symptoms (medium and high risk for AWS^a^)	No control group utilized	60±10	<>	31	<>	29	<>	Prior hospitalization for AWS: 31, multidrug use: 3	<>	Yale Alcohol Withdrawal Protocol, a benzodiazepine symptom-triggered treatment protocol paired with the mMINDS scoring system	<>
Gold et al. (retrospective cohort study) [17]	2007	Post-guideline period (07/2003- 05/2005)	Pre-guideline period (07/2000- 06/2002); all patients enrolled in the study were those who required either 200 mg of diazepam in 4 hours or an individual dose of 40 mg IV diazepam for control of agitation	45±1.2	45.7±1.8	41	54	41	54	Alcohol withdrawal seizure: 17, DTs: 40	Alcohol withdrawal seizure: 15, DTs: 53	<>	<>
Goodberlet et al. (retrospective study) [13]	2020	Post group receiving phenobarbital post guideline for severe AWS (09/2017 to 04/2018)	Pre group receiving BZD (09/2016 to 04/2017)	Median 52 (44-60)	Median 49.1 (37-59)	64 MICU (43%), SICU (21%)	68 MICU (28%), SICU (40%)	46	49	Alcohol use disorder: 57, AUD + severe withdrawal: 35, AUD + DTs: 20, AUD + seizures: 20, psychiatric disorder: 33, liver disease: 11	Alcohol use disorder: 61, AUD + severe withdrawal: 41, AUD + DTs: 15, AUD + seizures: 19, psychiatric disorder: 35, liver disease: 6	CAM-ICU and RASS scores used to assess agitation and depth of sedation, respectively. APACHE II score, MELD score	Same as the post group
Nguyen et al. (retrospective cohort study) [12]	2019	Phenobarbital + lorazepam	Lorazepam	54.2±10.8	48	36	51	31	46	Encephalopathy: 7, alcoholic cirrhosis: 8, pancreatitis: 3, malnutrition: 18. Seizures prior to admission: 6, history of alcohol withdrawal syndrome: 5	Encephalopathy: 7, alcoholic cirrhosis: 5, pancreatitis: 3, malnutrition: 15 seizures prior to admission: 6, history of alcohol withdrawal syndrome: 9	CIWA	CIWA
Oks et al. (retrospective observational study) [11]	2018	Phenobarbital group only	<>	48±12	<>	81	<>	79	<>	<>	<>	CIWA, RASS score	
Saukkonen et al. (abstract, retrospective study) [18]	2018	Phenobarbital group between 08/01/2016 and 10/04/2017	BZD group between 07/01/2015 and 07/15/2016	<>	<>	27	28	<>	<>	<>	<>	<>	<>
Shah et al. (retrospective study) [15]	2022	Front-loaded phenobarbital	Low intermittent phenobarbital	Median 48 (40-54)	Median 50 (42-55)	46	41	40	35	Prior AWS seizures: 13, other substance use disorder: 5, psychiatric illness: 12	Prior AWS seizures: 15, other substance use disorder: 9, psychiatric illness: 14	CIWA, RASS score	CIWA, RASS score
Tidwell et al. (retrospective cohort study) [14]	2018	Phenobarbital group	BZD group	45±11.4	52±15.5	60	60	44	43	Psychiatric disorder: 29, polysubstance abuse: 10, seizure disorder: 8, reactive airway disease: 6, liver disease: 16, previous DTs or withdrawal seizures: 27	Psychiatric disorder: 29, polysubstance abuse: 10, seizure disorder:5, reactive airway disease: 8, liver disease: 14, previous DTs or withdrawal seizures: 32	Dosages based on the presence or absence of DTs, previous history or no history of DTs	CIWA
Duby et al. (retrospective pre-post study) [16]	2014	Patients in the postintervention group (POST) were given escalating doses of diazepam and phenobarbital according to an AWS protocol	Patients in the preintervention group (PRE) were treated in a non-protocolized fashion and typically received continuous infusions or scheduled doses of BZDs per physician preference	50.7±13.8	55.7±8.7	75	60	81.3%	81.6%	History of alcohol withdrawal: 30.6%, history of psychosis: 12%, history of delirium tremens: 4%, history of seizures:21%	History of alcohol withdrawal: 40%, history of psychosis: 10%, history of delirium tremens: 10%, history of seizures: 18.3%	CIWA, RASS score, SOFA score	CIWA, RASS score, SOFA score

Baseline Characteristics and Comorbidities of Patients with AWS

According to Ammar et al., phenobarbital was administered to patients admitted to the surgical intensive care unit for the prevention or worsening of AWS [10]. In a study conducted by Oks et al., patients were treated with phenobarbital according to the pharmacological protocol of the medical intensive care unit to manage AWS [11]. For the treatment of AWS, Nguyen et al., Goodberlet et al., Saukkonen et al., and Tidwell et al., used benzodiazepine in the control group and phenobarbital in the experimental group [12-14,18]. According to Shah et al., patients in the control group received low intermittent phenobarbital, whereas patients in the experimental arm received front-loaded phenobarbital [15].

Duby et al. conducted a retrospective study where patients in the preintervention group (PRE) were treated in a non-protocolized fashion and typically received continuous infusions or scheduled doses of benzodiazepines according to physician preferences. The AWS protocol was used to administer escalating diazepam and phenobarbital to patients in the postintervention group (POST) [16]. As part of the retrospective cohort study conducted by Gold et al., patients who required escalating doses of benzodiazepines during the pre-guideline era (i.e., the control group) were admitted to intensive care units. While in the intensive care unit, they received benzodiazepine and phenobarbital as a non-protocolized treatment. In the post-guideline era, patients admitted to the ICU for the management of AWS were administered benzodiazepine and phenobarbital in a protocolized manner [17]. We found that the majority of the studies included in our study included patients with a prior history of AWS, polysubstance abuse, and alcohol withdrawal seizures [10,12-17].

Management and Monitoring Protocols Implemented in the ICU for the Management of AWS

The management protocols implemented for the treatment of AWS in an ICU setting in both control and experimental groups have been described in depth in Table 2. A variety of scoring systems were used for the assessment and monitoring of alcohol withdrawal. Ammar et al. implemented the Revised Clinical Institute Withdrawal Assessment for Alcohol (CIWA-Ar) scale, Simplified Acute Physiology Score (SAPS), Acute Physiology and Chronic Health Evaluation II (APACHE II), and Sequential Organ Failure Assessment (SOFA) score for the assessment and monitoring of alcohol withdrawal. Richmond Agitation and Sedation Scale (RASS) was implemented for the monitoring of sedation [10]. Oks et al., Nguyen et al., and Shah et al. used the CIWA score for the monitoring and assessment of patients admitted to the ICU for alcohol withdrawal [11,12,15]. APACHE II was the scoring system deployed by Goldy et al. for their study [17]. Goodberlet et al. implemented the APACHE II, Model for End-stage Liver Disease (MELD), and Confusion Assessment Method for Intensive Care Unit (CAM-ICU) scoring systems. RASS was deployed for monitoring of sedation [13]. Duby et al. used the SOFA score for the monitoring and assessment with RASS to monitor for sedation [16]. Apart from benzodiazepines and phenobarbital, a few other medications, particularly sedatives, were used for managing alcohol withdrawal patients both in the control and experimental arms of the studies included in our systematic review and meta-analysis. A few participants in the case series conducted by Ammar et al. received quetiapine, haloperidol, or a combination of the two for the management of alcohol withdrawal syndrome [10]. Patients requiring mechanical ventilation in the study conducted by Gold et al. received propofol [17]. Goodberlet et al. conducted a study where a few of the participants in both control and experimental arms received dexmedetomidine, clonidine, propofol, and antipsychotics [13]. A total of 11 patients in the experimental group and 26 patients in the control group received propofol and dexmedetomidine for the management of alcohol withdrawal syndrome in the study conducted by Saukkonen et al. [18]. A retrospective study conducted by Shah et al. mentions a few subjects, both in the control and experimental arm, requiring sedatives and other adjunctive medications (both ventilated and non-ventilated patients) such as dexmedetomidine, ketamine, propofol, antipsychotics and gabapentin [15]. Participants in the study conducted by Duby et al., required dexmedetomidine (4 patients in the experimental arm vs. 17 patients in the control arm), olanzapine (five patients in the experimental arm vs. seven patients in the control arm), haloperidol (4 patients in the experimental arm vs. 10 patients in the control arm) and quetiapine (two patients in the experimental arm vs. five patients in the control arm) for further management [16].

**Table 2:** Management protocols LD: loading dose; IM: intramuscular; h: hours; SD: standard deviation; %: percentage of patients; n: number of patients; PO: by mouth; TID: three times daily; BZD: benzodiazepine; CIWA-Ar: Revised Clinical Institute Withdrawal Assessment for Alcohol; APACHE II: Acute Physiology and Chronic Health Evaluation II; RASS: Richmond Agitation and Sedation Scale; CAM-ICU: Confusion Assessment Method for ICU; SOFA: Sequential Organ Failure Assessment; SAPS: Simplified Acute Physiology Score; MELD: Model for End-stage Liver Disease; AST: aspartate aminotransferase; ALT: alanine aminotransferase; MICU: medical intensive care unit ^a^Among the risk factors for sedation are age >65 years, hepatic dysfunction (AST and ALT levels two to three times the upper limit of normal), liver cirrhosis, head injuries, recent administration of opioids, recent administration of BZD within the past six hours, and recent administration of sedatives. Risk factors for respiratory compromise are pneumonia, rib fracture, chest tube, pulmonary contusion, cervical collar/spine brace. <> denotes not mentioned/not available.

Authors	Year	Dosing regimen		Reason for ICU admission		CIWA/additional scoring system/RASS score before the intervention, mean±SD/median (interquartile range)/(%)		BZD/BZD equivalent/haloperidol/antipsychotic/sedative-anesthetic use	
		Intervention	Control/comparative arm	Intervention	Control/comparative arm	Intervention	Control/comparative arm	Intervention	Control/comparative arm
Ammar et al. [10]	2020	Patients received a phenobarbital loading dose of 10-15 mg/kg ideal body weight, given as 3 separate doses of 40%, 30% and 30% of LD, 3 hours apart, followed by the taper maintenance dose. Patients with severe risk of AWS and low risk of sedation and respiratory (resp) compromise received LD of 15 mg/kg, those with severe risk of AWS, and high risk of sedation and resp compromise received 12 mg/kg, those at moderate risk of AWS and low risk of sedation received 12 mg/kg, those at moderate risk of AWS and high risk of sedation and resp depression received 10 mg/kg LD. LD was given as IM, while maintenance was given as IM or IV; MD was started at least 8 hours after LD and continued for 6 days, with a goal to maintain blood concentration of phenobarbital achieved by LD; BZD was discouraged post LD administration, while other sedatives were encouraged^a^		This was a single-center retrospective case series of patients who received phenobarbital monotherapy for the indication of AWS from August 1, 2018, to March 1, 2020. Critically ill adult patients (≥18 years old) who were admitted to the surgical/trauma ICU and received phenobarbital monotherapy for the prevention of worsening of AWS symptoms		CIWA 10 (6-10), SAPS score 24 (22-35), APACHE II 12 (9-16), SOFA score 3 (3-5), RASS score 0 (-1 to 0)	<>	One patient received a total of 20 mg lorazepam post–phenobarbital initiation over 5 days. Nine patients (29%) received nonbenzodiazepine adjunct therapy for agitation post–phenobarbital initiation, with 6 patients (19%) receiving quetiapine only, 7 (23%) receiving haloperidol only, and 4 (13%) receiving both quetiapine and haloperidol post–phenobarbital initiation.	<>
Gold et al. [17]	2007	Symptoms directed BZD to achieve a RASS score of 3-4; those requiring >100-200 mg BZD for agitation in an hour were given phenobarbital to achieve target sedation; >51% of patients received phenobarbital (median dose 390 mg)	17% received phenobarbital (260 mg median dose), half of whom required mechanical ventilation	The criteria for admission to the ICU, which remained constant throughout the study, were the requirement for either 200 mg of diazepam in 4 hours or an individual dose of 40 mg of intravenous diazepam for control of agitation		APACHE II 11.0±0.8	APACHE II 13.0±0.8	Propofol was the preferred sedative used for intubated patients, though only 17.8% required propofol	23% received propofol all of whom were already receiving mechanical ventilation
Goodberlet et al. [13]	2020	Patients eligible for phenobarbital LD were dosed using the divided dose strategy, where 40% of the total dose was given initially, 30% 3 hours later and the final 30% 3 hours after the second dose. Patients with a history of severe withdrawal or patients who showed signs and symptoms of withdrawal following the LD received phenobarbital taper. Taper begins on day 2 of therapy and continues for up to 6 days total, starting with 64.8 mg enterally twice daily and decreasing dose by 50% every 2 days	Symptoms directed scheduled BZD	Adult patients admitted to the medical/surgical/burn/trauma ICU were categorized into the PRE group if they had received scheduled benzodiazepines for at least 24 hours pre-guideline update (September 1, 2016-April 1, 2017), or to the POST group if they had received phenobarbital for severe alcohol withdrawal post-guideline update (September 1, 2017-April 1, 2018). Severe alcohol withdrawal was defined by at least one of the following criteria: alcohol withdrawal seizures on presentation, history of withdrawal seizures and at risk of withdrawal, delirium tremens on presentation, history of delirium tremens and at risk of withdrawal, or history of severe alcohol withdrawal		RASS >0 on day 1: 26 (40.6%), CAM-ICU positive on day 1: 32 (50%), MELD 7 (6-9), APACHE II 10 (5-13)	RASS >0 on day 1: 41 (60.3%) CAM-ICU positive on day 1: 24 (35.8%), MELD 6 (6-8), APACHE II 4 (3-9)	Patients with additional phenobarbital requirement: 11, use of dexmedetomidine on day 1: 28, use of clonidine on day 1: 3, use of propofol on day 1: 23, use of antipsychotics on day 1: 8	Patients with additional BZD requirement: 11, use of dexmedetomidine on day 1: 4, use of clonidine on day 1: 5, use of propofol on day 1: 4, use of antipsychotics on day 1: 14
Nguyen et al. [12]	2019	Patients admitted to ICU for AWS and was dosed with Lorazepam and Phenobarbital were enrolled	Patients who received >18 mg lorazepam in the ICU stay were enrolled	The study included patients aged ≥18 years who experienced AWS in the ICU, determined by an order for the CIWA protocol and diagnosis of AWS by the provider		CIWA score 10.3±7.2	CIWA score 13.8±7.4	<>	<>
Oks et al. [11]	2018	MICU pharmacological protocol advised administration of phenobarbital 130 mg every 15 min until patient achieved a RASS score of 0-1. All phenobarbital was administered IV	<>	Standard MICU policy mandated that patients with CIWA scores >15 despite therapy were admitted to the MICU		CIWA score 19±9	<>	<>	The mean benzodiazepine equivalent dose administered in 74 of 86 patient encounters prior to MICU admission was 23±25 mg
Saukkonen et al. [18]	2018	<>	<>	<>	<>	<>	<>	11 patients required propofol and dexmedetomidine	26 patients required propofol and dexmedetomidine
Shah et al. [15]	2022	Phenobarbital was used as an adjunct therapy if CIWA score remained above 20 despite 3 doses of 8 mg of IV lorazepam. Phenobarbital was used per protocol to achieve CIWA score of <20 for non-intubated and RASS score less than +2 for intubated patients. Front-loaded phenobarbital dose (10 mg/kg IV infusion over 30 min) from 07/2015 to 01/2017	Low intermittent phenobarbital dose (260 mg IV push × 1 followed by 130 mg IV push every 15 min as needed) from 01/2013 to 07/2015	Primary diagnosis of severe AWS who received phenobarbital according to the severe AWS protocol in place at the time of admission. Severe AWS was considered the primary diagnosis if AWS was the principal reason for the patient's admission to the hospital or to the ICU. Severe AWS was defined as a CIWA-Ar score ≥20. Patients were excluded if they were in intubated prior to phenobarbital administration or received phenobarbital doses not in accordance with the severe alcohol withdrawal protocol		CIWA score 24 (21-29)	CIWA score 24 (20-29)	Frequency of sedative infusions, n (%): all patients 33 (72), Intubated patients 13 (100), non-intubated patients 20 (61). Sedative infusion medication, n (%): lorazepam 32 (70), midazolam 1 (2.2), dexmedetomidine 9 (20), ketamine 12 (26) propofol 8 (17). Adjunctive medications, n (%): antipsychotics 25 (54), gabapentin 6 (13). Overall duration of any sedative infusion, h: mean (SD), 94 (73)	Frequency of sedative infusions, n (%): all patients 39 (95), Intubated patients 26 (100), non-intubated patients 13 (87). Sedative infusion medication, n (%): lorazepam 39 (95), midazolam 3 (7.3), dexmedetomidine 2 (4.9), ketamine 15 (37), propofol 13 (32). Adjunctive medications, n (%): antipsychotics 21 (51), gabapentin 2 (5). Overall duration of any sedative infusion, h: mean (SD), 88 (55)
Tidwell et al. [14]	2018	Active DT: 260 mg IV × 1 followed by 97.2 PO TID × 6, 64.8 mg PO TID × 6, 32.4 mg PO TID × 6 (tapering regimen). History of DT: 97.2 PO TID × 6 followed by 64.8 mg PO TID × 6, 32.4 mg PO TID × 6 (tapering regimen). No history of DT: 64.8 mg PO TID × 6 followed by 32.4 mg PO TID × 6 (tapering regimen); all patients received lorazepam 1 mg IV every hour as needed for agitation	Symptoms directed BZD according to the CIWA protocol	A retrospective cohort study at a 42-bed MICU in a private teaching hospital in Nashville, Tennessee. The study included medical ICU patients admitted from January 1, 2016, through June 30, 2017, and treated for the onset or prevention of AWS. A total of 147 patients were screened for eligibility, and 120 patients met inclusion criteria for participation in the study, with 60 receiving treatments with the phenobarbital protocol and 60 receiving treatments with the CIWA protocol		<>	<>	Dexmedetomidine use, no. of patients: 4; olanzapine use, no. of patients: 5; haloperidol use, no. of patients: 4; quetiapine use, no. of patients: 2; lorazepam equivalent used in ICU (mean±SD): 11.3±18	Dexmedetomidine use, no. of patients: 17; olanzapine use, no. of patients: 7; haloperidol use, no. of patients: 10; quetiapine use, no. of patients: 5; lorazepam equivalent used in ICU (mean±SD): 35.2±48.5
Duby et al. [16]	2014	Patients in the postintervention group (POST) were given escalating doses of diazepam and phenobarbital according to an AWS protocol. Escalating doses of phenobarbital (60 mg, 120 mg, 240 mg; route not indicated) after maximum 120 mg	Patients in the preintervention group (PRE) were treated in a non-protocolized fashion and typically received continuous infusions or scheduled doses of BZDs per physician preference	Patients were included if they were 18 years or older and admitted to an ICU between February 2008 and February 2010 (PRE) or between February 2012 and January 2013 (POST). A primary diagnosis of AWS was not required for study inclusion. However, patients were characterized according to the primary reason for ICU admission, that is, AWS or another diagnosis. Patients with all other comorbidities were deliberately included except for severe brain injury defined as a persistent Glasgow Coma Scale score of less than 8, because of the inability to fully assess withdrawal symptoms and sedation levels in these patients. Patients with a CIWA score of 8-20 (moderate to severe risk) and symptom-triggered escalating of load of BZD required ICU status		SOFA score 3.9±2.9, target RASS 0 to -2	SOFA score 6.1±3.7, target RASS 0 to -2	Patients received symptom-triggered doses of diazepam every 15-30 minutes until the target sedation level was achieved. Nurses were directed to continue escalating diazepam doses up to a maximum of 120 mg. Mean BZD usage in the POST group, 50-100 mg	Mean BZD usage in the PRE group, 300-350 mg

Results of the Meta-Analysis, Outcomes, and Complications

**Figure 2:**
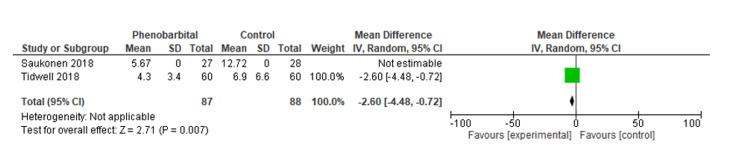
Forest plot of the length of the hospital stay for the phenobarbital vs. control group The green box represents the individual study effects and the black diamond represents the combined result of the studies. Source: [14,18]

**Figure 3:**
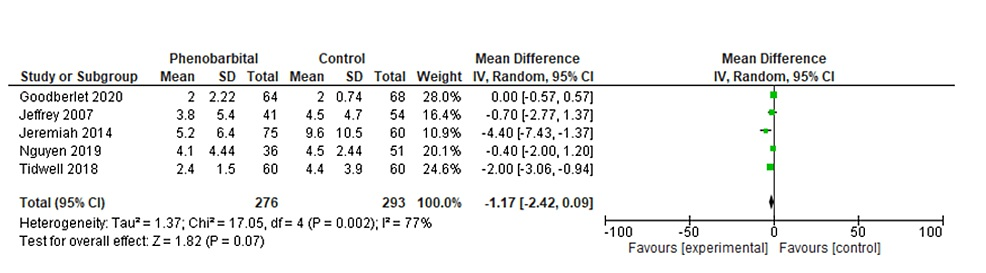
Forest plot of the length of ICU stay for the phenobarbital vs. control group The green box represents individual study effects and the black diamond represents the combined result of the studies. Source: [12-14,16,17]

**Figure 4:**
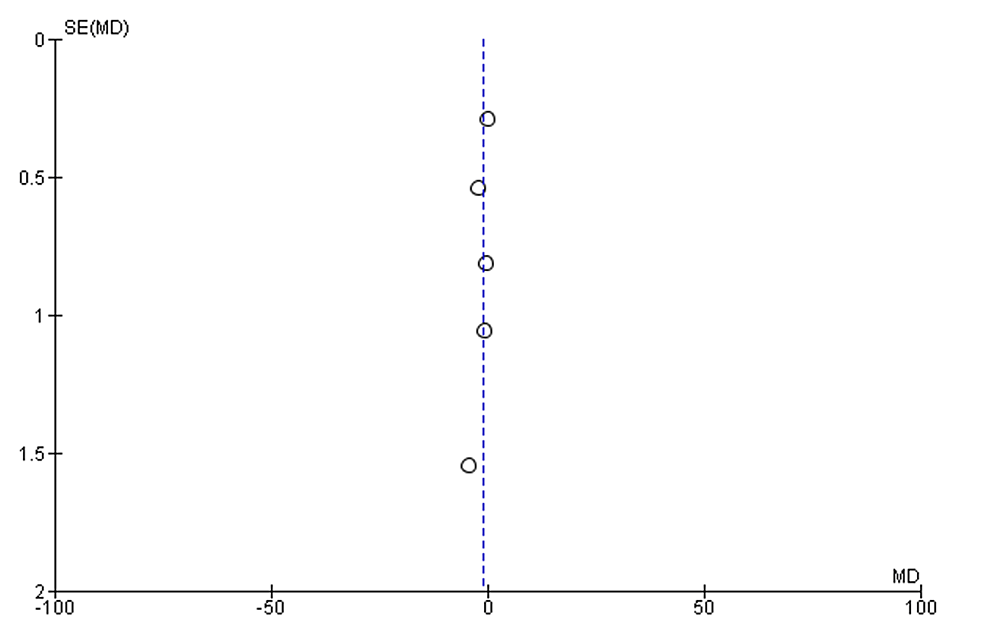
Funnel plot of the length of ICU stay for the phenobarbital vs. control group Circles represent standard errors for individual studies. Source: [12-14,16,17]

**Figure 5:**
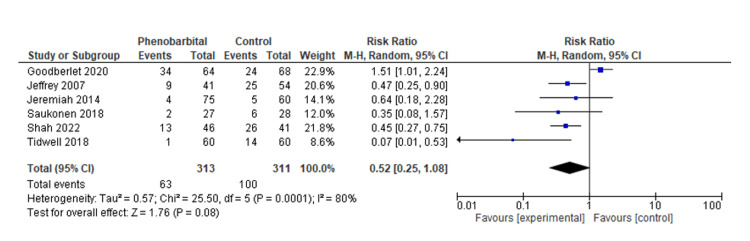
Forest plot of risk of intubation for the phenobarbital vs. control group The blue box represents individual study effects and the black diamond represents the combined result of the studies. Source: [13-18]

**Figure 6:**
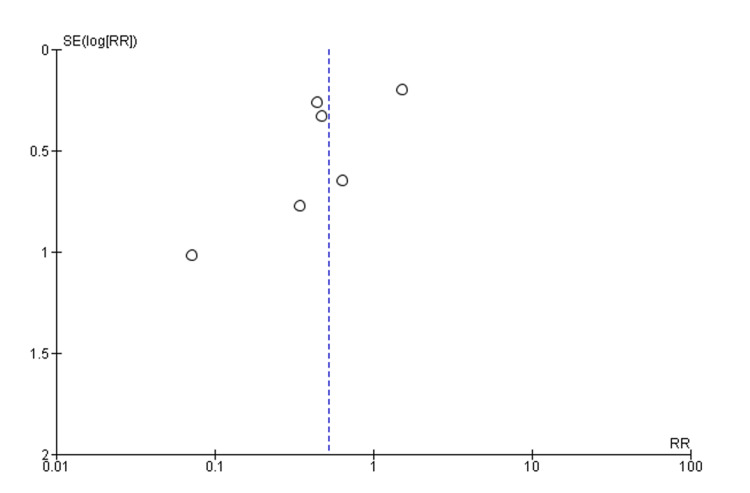
Funnel plot of risk of intubation for the phenobarbital vs. control group RR: relative risk Circles represent standard errors for individual studies. Source: [13-18]

Our meta-analysis included seven studies. The purpose of this study was to determine the length of hospital stay and ICU stay (days) as well as the rate of intubation (Tables 3, 4). Pooled analysis of the length of hospital stay included two studies with a total of 175 patients. The mean difference was statistically significant at -2.6 (95% CI, -4.48, -0.72, P=0.007). We were unable to comment on the heterogeneity in our meta-analysis due to the SD not being reported in one study (Figure 2) [14,18]. In the pooled analysis of ICU stay length, six studies with a total of 569 patients were included. Based on the results, there was no statistically significant difference regarding the length of stay in the intensive care unit, with a mean difference of -1.17 (95% CI, -1.17, 0.09, P=0.07), with considerable heterogeneity (I^2^=77%, P=0.002) (Figures 3, 4) [12-14,16-18]. We also examined the incidence of intubation between the experimental and control groups. The pooled analysis included six studies involving 624 patients. Phenobarbital vs. control had no significant differences in the incidence of intubation, with relative risk, or RR, 0.52 (95% CI, 0.25, 1.08, P=0.08), with considerable heterogeneity (I^2^=80%, P=0.0001) (Figures 5, 6) [13-18].

**Table 3:** Results for included studies n: number of patients; %: percentage of patients; h: hours; SBP: systolic blood pressure; DBP: diastolic blood pressure; HR: heart rate; bpm: beats per minute; IQR: interquartile range; SD: standard deviation; LD: loading dose; BZD: benzodiazepine; RASS: Richmond Agitation and Sedation Scale; CAM-ICU: Confusion Assessment Method for ICU; CIWA-Ar: Revised Clinical Institute Withdrawal Assessment for Alcohol; MICU: medical intensive care unit; APACHE II: Acute Physiology and Chronic Health Evaluation II ^a^Subgroup analysis: History of severe withdrawal, withdrawal seizures, or delirium tremens: median APACHE II score of 10 (5:13) in the POST guideline group as compared to 4 (2.8:25) in the PRE guideline group. Presentation with withdrawal seizures or delirium tremens: median APACHE II score of 10 (5:14.5) in the POST guideline group as compared to 5 (3.25:6.75) in the PRE guideline group. MICU patients: median APACHE II score of 10 (5:16) in the POST guideline group as compared to 6 (2:10) in the PRE guideline group. Phenobarbital load (mg/kg ideal body weight): median APACHE II score of 8 (4:13) when patients received a phenobarbital loading dose ≥9 mg/kg ideal body weight as compared to 10 (7:16) in patients receiving a loading dose of <9 mg/kg ideal body weight. ^b^The number of patients receiving dexmedetomidine was much lower in the phenobarbital group than in the CIWA group: 4 (7%) vs. 17 (28%), P=.002, with most patients receiving dexmedetomidine for symptom control while not receiving mechanical ventilation. ^c^Need for continuous sedation: in the POST group, 18 (24%) vs. 33 (55%), in the PRE group. <> denotes not mentioned/not available.

Authors	Year	Length (days) of hospital stay, mean±SD/median (Interquartile range)		Length (days) of ICU stay, mean±SD/median (Interquartile range)		Vitals/hemodynamics		Dosage of phenobarbital use, mean±SD/median (interquartile range)		CIWA/additional scoring system score/RASS after intervention		Duration of therapy	
		Intervention	Control/comparative arm	Intervention	Control/comparative arm	Intervention	Control/comparative arm	Intervention	Control/comparative arm	Intervention	Control	Intervention	Control/comparative arm
Ammar et al. [10]	2020	6 (4-15)	<>	2 (1-2)	<>	SBP (mmHg, mean±SD) improved from 146±13 to 139±17, DBP (mmHg, mean±SD) improved from 81±19 to 81±19, HR (bpm, mean±SD) improved from 98±23 to 92±18	<>	LD of 13 mg/kg ideal body weight	<>	RASS score -1 (-1 to 0)		Median duration of phenobarbital maintenance duration was 5 (3-6) days	<>
Gold et al. [17]	2007	<>	<>	3.8±5.4	4.5±4.7	<>	<>	390 (130-1430 mg)	260 mg (25%-75% quartile 87.5-650 mg)	The goal was to achieve a RASS score of 3-4	The goal was to achieve a RASS score of 3-4	<>	<>
Goodberlet et al. [13]	2020	8 (6-12)	4 (3-6)	2 (2-5)	2 (1-2)	<>	<>	Median loading dose: 9 mg/kg based on ideal body weight; taper was administered in 65.6% patients with median 4 days	Average duration of BZD therapy was 2.9 days; median was 3 days, more patients in the PRE group received BZD 24 hours prior to the start of treatment than the POST group (72% vs. 60%). The average total dose of lorazepam equivalent was 8.3 vs. 8.5 mg in the POST group^a^	CAM-ICU positive on day 1: 32 patients, RASS>0 on day 1: 26 patients	CAM-ICU positive on day 1: 24 patients, RASS>0 on day 1: 41 patients^a^	Median days 4 in 65.6% patients receiving taper	2.9 days (mean)
Nguyen et al. [12]	2019	<>	<>	4.1 (2.4-8.4)	4.5 (2.8-6.1)	<>	<>	35.5±48.8 mg lorazepam, 909.4±785.4 mg phenobarbital	48.2±28.0 lorazepam	CIWA score (mean±SD) 1.8 ± 9.0	CIWA score (mean±SD) 6.5±8.5	Median (IQR) 2.7 (1.7-6.4)	Median (IQR) 3.1 (1.6-4.8)
Oks et al. [11]	2018	10±6	<>	5±3	<>	<>	<>	1978±1532 mg (median 1548), corresponding to 25±17 mg/kg ideal body weight	<>	CIWA score could not be obtained due to the loss of paperwork. The achievement of a RASS score of 0 to -1 defined the efficacy of phenobarbital for the treatment of AWS		5.2±2.9 (mean±SD)	<>
Saukkonen et al. [18]	2018	Mean: 5.67	Mean: 12.73	Mean: 1.9	Mean: 6.24	<>	<>	<>	<>			<>	<>
Shah et al. [15]	2022	<>	<>	Median (IQR), intubated patients: 193 (170-206) h, non-intubated patients: 96 (59-124) h. Time to start of infusion to target sedation: 2.7 (1.0-7.5) h median)	Median (IQR), intubated patients: 156 (106-189) h, non-intubated patients: 162 (127-214) h. Time to start of infusion to target sedation: 2.0 (1.0- 2.7 h median)	<>	<>	Phenobarbital cumulative dose: median 1200 mg (111-1585), 15 (10-20) mg/kg, median phenobarbital serum level: median 14 (12-16) mcg/mL, lorazepam requirements, median (IQR): pre-phenobarbital 36 (20-46) mg, post- phenobarbital 86 (24-297)	Phenobarbital cumulative dose: median 1170 mg (910-1300), 15 (12-20) mg/kg, median phenobarbital serum level: median 15 (12-20) mcg/mL, lorazepam requirements, median (IQR): pre-phenobarbital 58 (41-80) mg, post- phenobarbital 228 (115-298) mg	Control of AWS was defined as a CIWA-Ar score <20 for non-intubated patients or a RASS score less than +2 for intubated		Time from phenobarbital administration to control of AWS, h: median (IQR) 2.7 (1.0-7.5), overall duration of any sedative infusion, h: mean (SD) 94 (73)	Time from phenobarbital administration to control of AWS, h: median (IQR) 2.0 (1.0-2.7), overall duration of any sedative infusion, h: mean (SD) 88 (55)
Tidwell et al. [14]^b^	2018	4.3±3.4	6.9±6.6	2.4±1.5	4.4±3.9	<>	<>	<>	<>			<>	<>
Duby et al. [16]	2014	<>	<>	5.2±6.4	9.6±10.5	<>	<>	Mean phenobarbital usage, 50-100 mg	Mean phenobarbital usage, 0-50 mg	Target RASS score of 0 to -2	Target RASS score of 0 to -2	Duration of sedation (days, mean±SD): 3.5±3.5^c^	Duration of sedation (days, mean±SD): 10.8±8.9^c^

**Table 4:** Complications COPD: chronic obstructive pulmonary disease; MICU: medical intensive care unit; IQR: interquartile range; SD: standard deviation ^a^There was a significant reduction in the use of mechanical ventilation post-guideline (21.9% vs. 47.3%; P=.008). Those requiring mechanical ventilation post-guideline had a higher ICU length of stay (6.4±1.6 vs. 3.1±1.3 days; P=.001) and incidence of nosocomial pneumonia (55.5% vs. 12.5%; P=.02). ^b^Time on mechanical ventilation, days (median): 2 (1:4) in the POST vs. 1 (1:2) in the PRE group. ^c^Reasons for mechanical ventilatory support (number of patients): obtundation with loss of airway clearance before phenobarbital administration (6), UGI bleeding with hemodynamic instability and/or need for airway protection during upper endoscopy (6), seizure with loss of airway protection (1), aspiration pneumonia preceding MICU admission (1), cardiac arrest of unclear etiology in MICU (1), COPD with hypercarbic respiratory failure (1), progressive hepatic encephalopathy (1). No patient was intubated due to phenobarbital use alone. ^d^Ventilator-free days in the POST group 26.3±5.6 vs. 21.3±9.5 in the PRE group. <> denotes not mentioned/not available.

Authors	Year	Intubation		Bradycardia		Hypotension		Mortality		Other	
		Intervention	Control/comparative arm	Intervention	Control/comparative arm	Intervention	Control/comparative arm	Intervention	Control/comparative arm	Intervention	Control/comparative arm
Ammar et al. [10]	2020	3 patients during phenobarbital administration	<>	<>	<>	3	<>	0	<>	0 patients developed respiratory depression or oversedation	<>
Gold et al. [17]	2007	21.9% of the patients in the arm^a^	47.3% of the patients in the arm^a^	<>	<>	<>	<>	<>	<>	Nosocomial pneumonia and complications of the infection: 19.5% of the patients	Nosocomial pneumonia and complications of the infection: 30.9% of the patients
Goodberlet et al. [13]	2020	34 patients (53.1%)^b^	24 patients (35.3%)^b^	<>	<>	<>	<>	ICU mortality: 1 patient (1.6%), hospital mortality: 5 patients (7.8%)	ICU mortality: 1 patient (1.5%), hospital mortality: 1 patient (1.5%)	Seizures after treatment initiation: 2 patients	Seizures after treatment initiation: 2 patients
Nguyen et al. [12]	2019	0 patient	3 patients	0	0	0	0	0	0	0	0
Oks et al. [11]	2018	17 patients^c^	<>	<>	<>	<>	<>	<>	<>	<>	<>
Saukkonen et al. [18]	2018	2 patients	6 patients	<>	<>	<>	<>	<>	<>	<>	<>
Shah et al. [15]	2022	13 patients, duration of mechanical ventilation (vent), median (IQR), h: 124 (98-147)	26 patients, duration of mechanical vent median (IQR), h: 109 (87-127)	<>	<>	10	23	<>	<>	<>	<>
Tidwell et al. [14]	2018	1 patient	14 patients	<>	<>	<>	<>	<>	<>	<>	<>
Duby et al. [16]	2014	4 patients (5%), time on mechanical ventilation, mean (SD), days: 1.31±5.6^d^	13 patients (22%), time on mechanical ventilation, mean (SD), days: 5.6±13.9^d^	<>	<>	<>	<>	7 patients (12%)	2 patients (3%)	<>	

Risk of Bias of Included Studies

On the NIH risk-of-bias assessment tool, all of the studies included scored 10 or above, except for the abstract (a retrospective study) by Saukkonen et al., which received a score of 5 [11-18]. On the NIH tool, the case series included in our study received an overall score of 8 out of 9 [10].

Discussion

The purpose of our meta-analysis was to evaluate the effectiveness of phenobarbital in the treatment of alcohol withdrawal syndrome. Our study examined the patient's length of hospital stay, length of stay in the intensive care unit, and interventions such as intubations during hospitalization for alcohol withdrawal syndrome. Based on our analysis, the mean difference in hospital stay was statistically significant at -2.6 (95% CI, -4.48, -0.72, P=0.007) compared to the benzodiazepine group. We were unable to comment on the heterogeneity in our meta-analysis due to the SD not being reported in one study [14-18]. Based on the results of our meta-analysis, there was no statistically significant difference in the length of stay in the intensive care unit compared to the control/comparative arm, with a mean difference of -1.17 (95%CI, -1.17, 0.09, P=0.07), and considerable heterogeneity (I^2^=77%, P=0.002) [12-14,16,17]. Our meta-analysis also investigated the risk of intubation between the phenobarbital and the control/comparative group. There was a statistically significant difference in the incidence of intubation, RR 0.52 (95% CI, 0.25, 1.08, P=0.08), with considerable heterogeneity (I^2^=80%, P=0.0001) [13-18].

Only two studies with a total population of 175 participants compared the mean length of hospital stay between the intervention and the control arm whereas only one study compared the median length of hospital stay between the intervention and the control arm [13,14,18]. Unfortunately, due to the lack of SD values, the heterogeneity of the data could not be evaluated. Both Tidwell et al. and Saukkonen et al. found that phenobarbital treatment reduced hospital stay in both arms. We also found the same result in our analysis. The small population of the subgroup is a limitation of the analysis [14,18]. In contrast, Goodberlet et al. reported the opposite findings showing more prolonged hospitalization in the phenobarbital group compared to benzodiazepine [13].

ICU stay was reported in all the studies included in the systematic review [10-18]. Saukkonen et al. confirmed a difference in the length of stay in the ICU without mentioning a standard deviation in their study. The finding by Saukkonen et al. was promising as it demonstrated significantly fewer days of stay (1.91 days in the phenobarbital arm vs. 6.24 days in the benzodiazepine arm). As Saukkonen et al. only published a conference abstract, no meaningful data could be gathered to identify the cause of these contradictory findings [18].

Tidwell et al. also reported a significant reduction in the length of the ICU stay in the phenobarbital arm [14]. According to Duby et al., the ICU stay was also reduced in the phenobarbital group [16]. According to Goodberlet et al., patients who received phenobarbital spent longer in the intensive care unit. The extended ICU stay can be attributed to the study limitations since patients in the phenobarbital arm had severe symptoms, which could explain the prolonged stay in the ICU. In addition, patients were selected only when dexmedetomidine administration was limited in the institute, thereby influencing the choice of sedatives and other pharmacological agents [13].

We also examined interventions such as intubation. According to the study by Tidwell et al., the phenobarbital arm showed a decreased incidence of intubation [14]. Conversely, none of the five other studies were able to demonstrate such a link. We also found that there was no significant correlation between phenobarbital administration and lower intubation rates when compared to the control group in our meta-analysis. Reduced sedation needs may be responsible for the lower incidence of intubations, as previously reported [13-18].

Additionally, adverse effects such as bradycardia, hypotension, and mortality were examined. The only study to record bradycardia and hypotension was that of Nguyen et al., which found no incidence in the parameters of either arm [12]. The remaining studies did not include these parameters. Monitoring and documentation were not as effective as they could have been because the included studies were retrospective. The limitations of these studies include the inability to control the acquisition of the desired data, which may lead to the loss of essential details. The study by Shah et al. showed that front-loaded phenobarbital participants experienced significantly fewer episodes of hypotension than those receiving low intermittent dosing of phenobarbital [15]. As reported by Goodberlet et al., there was no difference in mortality between the two arms [13]. Contrary to this, Duby et al. reported improved mortality outcomes in patients treated with phenobarbital [16]. Additionally, phenobarbital used for epilepsy is easily measurable, giving it an advantage over benzodiazepines. Therefore, phenobarbital is unlikely to cause adverse effects [19].

Some studies have been evaluated in the previous meta-analyses in recent years. Hammond et al. reviewed nine studies in 2016 and found that the phenobarbital group had lower hospital stays, fewer ICU admissions, and decreased mechanical ventilation as compared to the benzodiazepine group [20]. However, the study was limited to 294 patients, posing a serious limitation. A systematic review by Mo et al. found barbiturates to be superior to benzodiazepines in cases of severe AWS or benzodiazepine resistance [21].

Limitations

We have identified a major limitation of our analysis as the inclusion of retrospective studies. This increases the possibility of selection bias and limits the observer's control over documentation and vital signs' monitoring. The relatively small size of our study is another limitation of our analysis. The analysis was limited by the fact that a few critical parameters, such as hospital stay, were reported in only three studies, making it difficult to reach meaningful conclusions. Additionally, no study has focused on the safety profile of phenobarbital. Also, some studies did not provide data on adverse effects. A final problem was the lack of uniform rules or guidelines for administering phenobarbital, which led to disparities between the results of different studies.

## Conclusions

In conclusion, phenobarbital can help reduce the length of hospital stay. However, it does not seem to have an impact when it comes to the length of ICU stay and the risk for intubation. Various studies have reported contradictory results. Since some vital details are lacking in the included studies, it is important to mention the need for large-scale, multi-center studies to be conducted before solid conclusions and recommendations can be made. Another important chapter that is missing from the previous studies is the safety of phenobarbital and the uniformity of dosing. Drug administration should also be adapted according to the severity of the symptoms. Additionally, there is a lack of detailed research regarding the adverse effects of phenobarbital administration in the context of alcohol withdrawal syndrome. Accordingly, the current evidence supports recommending phenobarbital for the treatment of alcohol withdrawal syndrome at a broad level.
